# *Lactobacillus plantarum* ATG-K2 and ATG-K6 Ameliorates High-Fat with High-Fructose Induced Intestinal Inflammation

**DOI:** 10.3390/ijms22094444

**Published:** 2021-04-24

**Authors:** Miey Park, Eun-Jung Park, So-Hyeun Kim, Hae-Jeung Lee

**Affiliations:** 1 Institute for Aging and Clinical Nutrition Research, Gachon University, Seongnam-si 13120, Korea; mpark@gachon.ac.kr (M.P.); ejpark@gachon.ac.kr (E.-J.P.); sohun94@naver.com (S.-H.K.); 2 Department of Food and Nutrition, Gachon University, Seongnam-si 13120, Korea

**Keywords:** obesity, small intestine, inflammation, *Lactobacillus* spp.

## Abstract

Obesity has become a worldwide health problem, and many significant inflammatory markers have been associated with the risk of side effects of obesity and obesity-related diseases. After a normal diet or high-fat diet with high-fructose water (HFHF) for 8 weeks, male Wistar rats were divided randomly into four experimental groups according to body weight. Next, for 8 weeks, a normal diet, HFHF diet, and HFHF diet with *L. plantarum* strains ATG-K2 or ATG-K6 were administered orally. Compared to the control group, the HFHF diet group showed significantly increased visceral fat, epididymal fat, and liver weight. The mRNA and protein expression levels of FAS and SREBP-1c were higher in the HFHF diet group than in the HFHF diet with *L. plantarum* strains ATG-K2 and ATG-K6. The HFHF diet with *L. plantarum* strain ATG-K2 showed significantly decreased inflammatory cytokine expression in the serum and small intestine compared to the HFHF diet group. Furthermore, histological morphology showed minor cell injury, less severe infiltration, and longer villi height in the small intestine ileum of the HFHF diet with *L. plantarum* strains groups than in the HFHF diet group. These results suggest that *L. plantarum* strains K2 and K6 may help reduce intestinal inflammation and could be used as treatment alternatives for intestinal inflammatory reactions and obesity.

## 1. Introduction

Obesity rates have increased worldwide in conjunction with chronic illnesses. Obesity is associated with low-grade systemic inflammation, which is considered a precursor of various degenerative disorders [1,2]. This inflammation is a significant cause of insulin resistance related to the development of type 2 diabetes (T2D) when no large-scale tissue damage has occurred, accompanied neither by infection nor signs of autoimmunity [3,4,5]. Responding to excess calories results in adipose tissue modification, leads to chronic inflammation, and orchestrates a pro-inflammatory cytokine response by releasing various cytokines into systemic circulation [4,6].

The small intestine has a gatekeeping function in the physical intermediate stage between the body and the diet [7]. The intestinal mucosal barrier is embedded with many immune cells and antimicrobial peptides, maintaining intestinal homogeneity by and antigens must be maintained [8]. The intestinal mucus layer contains antimicrobial peptides, which limit the number of bacteria and serve as the first line of immune defense [9,10]. This function is critical to health, and it has been proposed that barrier dysfunction contributes to both intestinal and systemic diseases [8,11].

Cytokines and chemokines, essential for intercellular communication, support gut mucosal homeostasis and are a leading cause of intestinal inflammation and inflammation-associated damage [8,12,13]. Obesity promotes inflammatory changes in intestinal immune cell populations and is associated with changes in the intestinal microflora [14,15,16]. The mucosal immune system and intestinal microbiota are greatly affected by dietary fiber and short microbial chain fatty acids (SCFAs), which have anti-inflammatory effects [17]. Dietary fiber deficiencies result in the erosion of the intestinal mucus barrier, and a long-term shortage of dietary fiber is associated with an increase in mucin-dissolving bacteria such as *Akkermansia muciniphila* [18]. High-fiber supply facilitates the release of SCFAs and leads to the secretion of IL-18, which is involved in the repair of the intestinal mucosal barrier [17,19].

Several studies have reported that administration of *Lactobacillus spp*. reduces diet-induced body weight gain and decreases blood glucose and insulin levels [20,21,22]. Moreover, after oral administration for 8 weeks, *Lactobacillus spp*. reduced inflammation and increased oxidative phosphorylation in white adipose tissue [23]. However, few studies have shown that the decrease in the inflammatory response of the small intestine due to *Lactobacillus spp.* administration improves obesity over time. Accordingly, in this study, we assessed the effect of intake of *Lactobacillus plantarum* isolated from Korean fermented cabbage on reducing inflammatory reactions in the small intestine. Our results provide significant insights into *Lactobacillus plantarum* strains as potential alternatives for treatment of intestinal inflammatory reactions and obesity.

## 2. Results

### 2.1. Effect of L. plantarum K2 and K6 on Morphological Changes in the High-Fat Diet Rat Model

**Figure 1:**
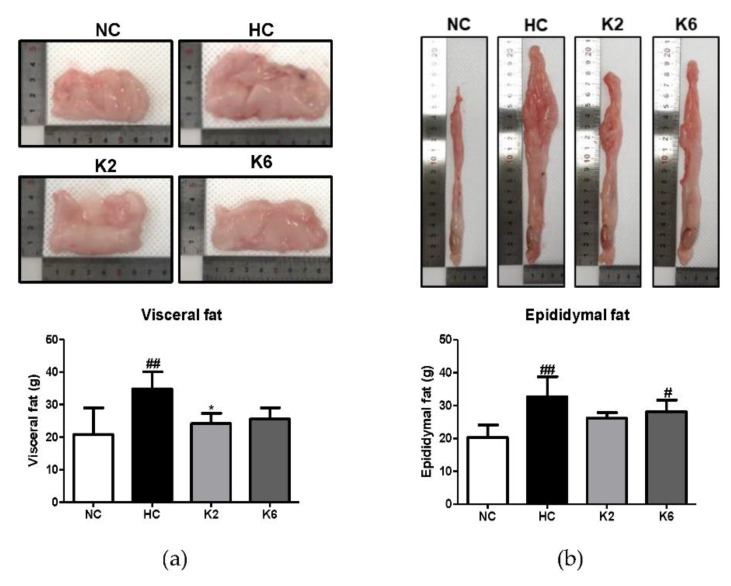

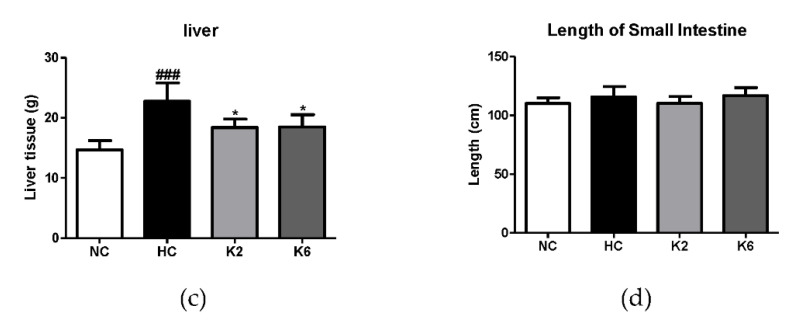
Effect of *L. plantarum* treatments on organ weight and small intestine length. Visceral fat (**a**) and epididymal fat (**b**) were evaluated for weight and size. Liver tissue (**c**) was also evaluated for weight and small intestine (**d**) for length. Values represent the mean ± SEM. # *p* < 0.05, ## *p* < 0.01, and ### *p* < 0.001 vs. control group (NC), * *p* < 0.05 vs. HC group. NC, normal diet control; HC, HFHF diet control; K2, HFHF diet with K2; K6, HFHF diet with K6.

To assess the effect of *L. plantarum* K2 and K6 on morphological changes, we measured the fat and liver weight and length of the small intestine. As shown in Figure 1a, the visceral fat weight of the HC group was higher than that of the NC group, and the weight of visceral fat that was increased by HC was significantly reduced by treatment with K2. Epididymal fat weight followed a pattern of HC > K6 > K2 > NC (Figure 1b). Both K2 and K6 showed significant changes in liver weight, and small intestine length did not change between the groups (Figure 1c,d).

### 2.2. Effect of L. plantarum K2 and K6 on Lipogenesis in Small Intestine

**Figure 2:**
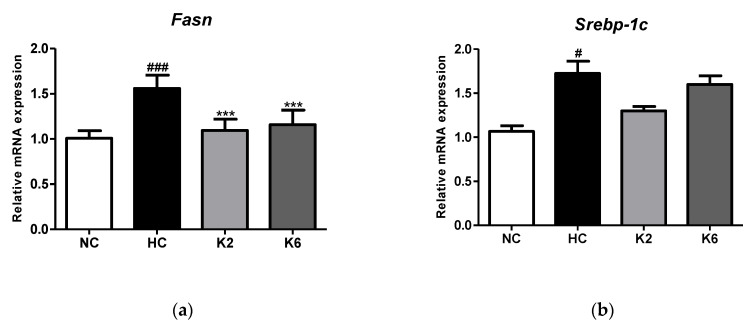
Effect of *L. plantarum* treatments on mRNA expression of lipogenesis-related genes in the HFHF diet rat. mRNA expression level of (**a**) *Fasn* and (**b**) *Srebp-1c* in the small intestine. Values represent the mean ± SEM. # *p* < 0.05 and ### *p* < 0.001 vs. control group (NC), *** *p* < 0.001 vs. HC group. NC, normal diet control; HC, HFHF diet control; K2, HFHF diet with K2; K6, HFHF diet with K6; *Fasn*, fatty acid synthase; *Srebp-1c*, sterol regulatory element-binding protein 1c.

For the understanding of the effect of *L. plantarum* K2 and K6 on HFHF diet induced small intestine lipogenesis, we first investigated the mRNA expression of lipogenesis markers. As shown in Figure 2, oral administration of *L. plantarum* ATG-K2 and ATG-K6 reduced *Fasn* and *Srebp-1c* mRNA expression in the small intestine of HFHF diet-fed rats. As shown in Figure 2a, the HFHF diet led to an increase in relative mRNA expression in the HC group, while oral administration of *L. plantarum* K2 and K6 significantly lowered the mRNA expression levels of FAS (*p* < 0.001) in the small intestine of the HFHF diet rats.

### 2.3. Effect of the L. plantarum K2 and K6 on Inflammation and Cytokine

**Figure 3:**
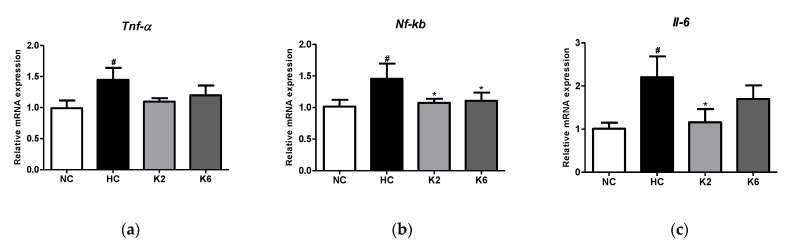
Effect of *L. plantarum* treatments on mRNA expression of inflammatory-related genes in the HFHF diet rat. mRNA expression level of (**a**) *Tnf-α*, (**b**) *Nf-κb*, and (**c**) *Il-6* in the small intestine. Values represent the mean ± SEM. # *p* < 0.05 vs. control group (NC), * *p* < 0.05 vs. HC group. NC, normal diet control; HC, HFHF diet control; K2, HFHF diet with K2; K6, HFHF diet with K6; *Tnf-α*, tumor necrosis factor-alpha; *Nf-κb*, nuclear factor-kappa B; *Il-6*, interleukin-6.

**Figure 4:**
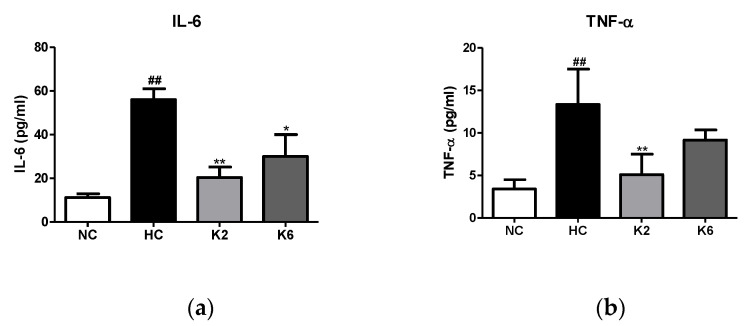
Effect of *L. plantarum* treatments on the production of cytokines of small intestine mucosa in the HFHF diet rat. Production concentrations of (**a**) IL-6 and (**b**) TNF-α in the culture supernatant. Values represent the mean ± SEM. ## *p* < 0.01 vs. control group (NC), * *p* < 0.05 and ** *p* < 0.01 vs. HC group. NC, normal diet control; HC, HFHF diet control; K2, HFHF diet with K2; K6, HFHF diet with K6; IL-6, interleukin-6; TNF-α, tumor necrosis factor-alpha.

**Figure 5:**
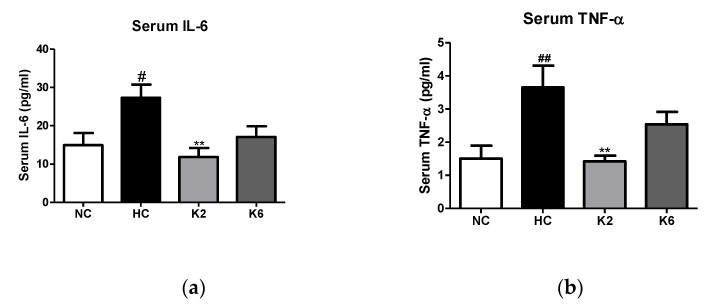
Effect of *L. plantarum* treatments on the production of cytokines in serum in the HFHF diet rat. Production concentrations of (**a**) IL-6 and (**b**) TNF-α in the serum. Values represent the mean ± SEM. # *p* < 0.05 and ## *p* < 0.01 vs. control group (NC), ** *p* < 0.01 vs. HC group. NC, normal diet control; HC, HFHF diet control; K2, HFHF diet with K2; K6, HFHF diet with K6; IL-6, interleukin-6; TNF-α, tumor necrosis factor-alpha.

To assess the effect of L. plantarum K2 and K6 strains on intestinal inflammation, we measured the levels of cytokines in the small intestine. As shown in Figure 3, the Tnf-α, *Nf-κb* and *Il-6* levels in the HC group were markedly higher than those in the NC group (*p* < 0.05). The mRNA expression levels of inflammatory cytokines in the small intestine of rats orally administered *L. plantarum* K2 and K6 decreased significantly compared to those in the HC group (*p* < 0.05). In addition, we evaluated the levels of inflammatory cytokines in the small intestinal mucosa of rats fed an HFHF diet. As shown in Figure 4, the HC group showed significantly (*p* < 0.01) higher concentrations of IL-6 and TNF-α compared to the NC group, while the levels of IL-6 and TNF-α in the oral administration of *L. plantarum* K2 significantly (*p* < 0.01) decreased compared to those in the HC group. This result is in line with the inflammatory cytokine results in the serum (Figure 5).

### 2.4. Effect of L. plantarum K2 and K6 on Protein Expression in Small Intestine

**Figure 6:**
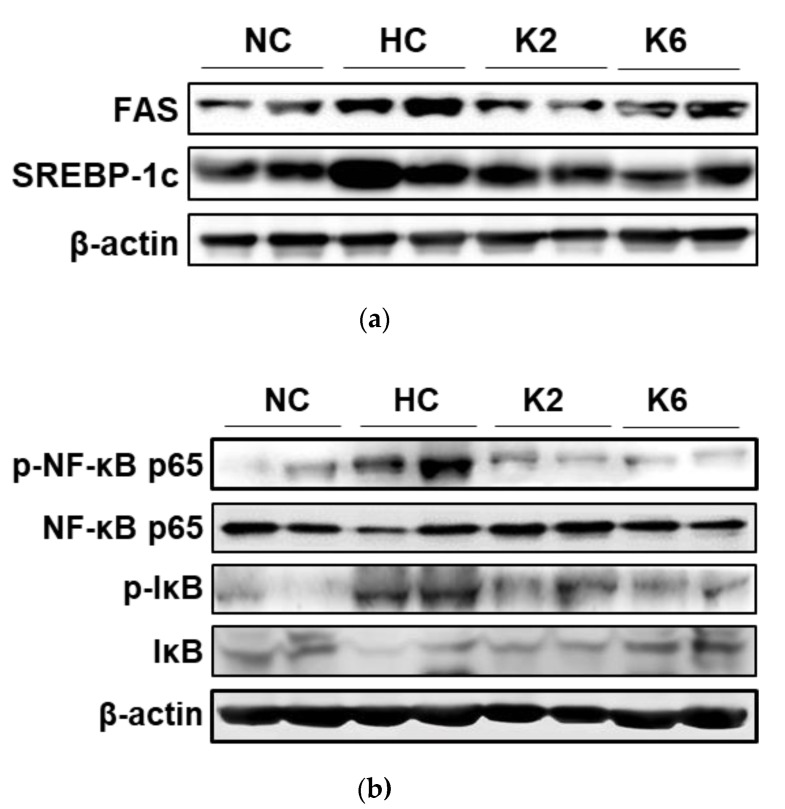
Effect of *L. plantarum* treatments on protein expression of lipogenesis and adipogenesis markers, and NF-κB activation in the HFHF diet rat. Protein expression level of FAS, SREBP-1c, and PPARγ (**a**) and phosphorylation level of NF-κB and IκB (**b**) in the small intestine. Images are representative of at least 3 independent experiments (for each group *n* = 2). NC, normal diet control; HC, HFHF diet control; K2, HFHF diet with K2; K6, HFHF diet with K6; FAS, fatty acid synthase; SREBP-1c, sterol regulatory element-binding protein 1c; p-NF-κB p65, phospho-nuclear factor-kappa B p65; NF-κB p65, nuclear factor-kappa B p65; p-IκB, phospho-inhibitors of kappa B; IκB, inhibitors of kappa B.

To demonstrate the effect of *L. plantarum* K2 and K6 on protein expression in the small intestine, we performed the western blot analysis. In the small intestine, FAS and SREBP-1c protein expression was higher in the HC group than in the NC group (Figure 6a). In addition, the expression levels of phospho-NF-κB p65 (p-NF-κB p65) and phospho-IκB (p-IκB) in the HFHF diet rats were higher than those in the NC group. In contrast, decreased phosphorylation levels of p-NF-κB p65 and p-IκB were observed in groups orally administered *L. plantarum* K2 and K6 compared to those in the HFHF diet rats (Figure 6b).

### 2.5. Effect of L. plantarum K2 and K6 on Histological Morphology in Small Intestine

**Figure 7:**
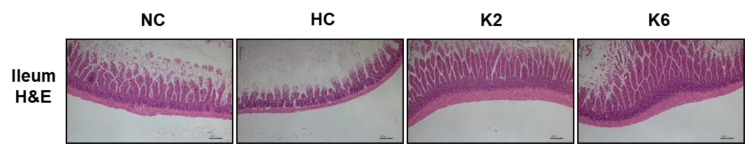
Effect of *L. plantarum* treatments on the histological morphology of the ileum in the HFHF diet rat. NC, normal diet control; HC, HFHF diet control; K2, HFHF diet with K2; K6, HFHF diet with K6; H&E, Hematoxylin and eosin staining. The scale bar shows 200 μm.

As shown in Figure 7, the HC group showed intestinal lesions and destruction of the epithelium of the small intestine ileum compared to that of the NC group in hematoxylin-eosin staining (40X). In addition, histological changes showed a decrease in villi height in the HC group compared to the control group. Compared with the HC group, minor cell injury and less severe infiltration of inflammatory cells in the submucosa were observed after oral administration of *L. plantarum* K2 and K6.

## 3. Discussion

Various factors cause inflammatory responses, and appropriate reactions have beneficial effects, while excessive inflammation leads to severe tissue damage [24,25]. Obesity is a critical factor that causes inflammation [26,27]. Consumption of oily foods continually produces triglycerides, and adipocytes start to die, thereby activating immune cells, causing inflammatory conditions such as fatty liver disease and inflammatory bowel disease [28,29]. Several studies have reported that consumption of lactobacilli is associated with decreased body fat [30,31]. *Lactobacillus gasseri* BNR17 (BNR17), isolated from human breast milk, inhibited increases in adipocyte tissue weight in high-sucrose diet-fed Sprague-Dawley rats [30]. Oral treatment with L. plantarum Y44 and a high-fat diet decreased the epididymal adipocyte area compared with that of the HFD group [31]. The present study demonstrated and confirmed that *L. plantarum* K2 and K6 have anti-obesity effects in rats like other lactobacilli.

In this study, the HFHF diet increased expression of lipogenesis markers FAS and SREBP-1c in the small intestine compared to the normal-diet rats. Oral treatment with *L. plantarum* ATG-K2 and ATG-K6 reduced expression of these markers in the small intestine, confirming previous findings that *L. plantarum* ATG-K2 and ATG-K6 affect the liver [32]. Accumulating evidence has revealed that administration of lactobacilli reduces obesity and decreases the inflammatory response of the small intestine by lactobacilli, though the underlying mechanisms are still not understood. To the best of our knowledge, this study is the first to demonstrate the effects of *Lactobacillus plantarum* strains ATG-K2 and ATG-K6 on HFHF diet-induced small-intestine inflammation.

The small intestine is a long digestive organ responsible for the breakdown and absorption of ingested food [33]. Inflammation of the small intestine can be accompanied by abdominal pain and bowel movement [34]. Intake of high-fat foods has been reported to induce changes in the intestinal flora, thereby increasing intestinal permeability, a significant factor in the onset of Crohn’s disease [35]. NF-κB, a critical transcriptional regulator of the inflammatory response, is also a crucial factor in intestinal inflammation [36] and is activated by various stimuli, including inflammatory cytokines such as TNF and pathogen-derived molecules [37]. In the present study, we demonstrated that administration of *Lactobacillus plantarum* strains ATG-K2 and ATG-K6 reduced HFHF diet-induced small-intestine inflammation by regulating pro-inflammatory cytokines in animal models. Both mRNA and protein expression levels of inflammatory marker NF-κB in the small intestine were significantly increased in the HC group compared to the NC group, while expression levels of inflammatory markers in the K2 and K6 groups significantly decreased. Other markers, TNF-α and IL-6, showed a tendency to decrease in the *Lactobacillus*-treated groups compared to the HC group.

In summary, the present study demonstrated that *Lactobacillus plantarum* strains ATG-K2 and ATG-K6 are effective in decreasing intestinal lipid accumulation and inflammation in HFHF diet rats by modulating serum and intestinal biomarkers. Therefore, our results revealed that *Lactobacillus plantarum* strains ATG-K2 and ATG-K6 could be used as treatment alternatives for intestinal inflammatory reactions and obesity. Further studies considering the practical approach are needed to investigate the optimal dose of *Lactobacillus* and the best way to deliver the strains to the intestine.

## 4. Materials and Methods

### 4.1. Animals and Bacterial Administration

All animal procedures were approved by the Institutional Animal Care and Use Committee of Gachon University (GIAUAC -R2019014). Six-week-old male Wistar rats were obtained from Orient Bio Co. (Seongnam, Korea). After adaptation for one week, rats were given free access to food and water and were fed a regular diet (ND, D12450B; Research Diets, New Brunswick, NJ, USA) or a high-fat diet (45% of calories from fat; D12451, Research Diets, New Brunswick, NJ, USA) with 10% fructose in the drinking water (HFHF). After 8 weeks, the rats were randomly divided into four groups (*n* = 7, each) according to body weight: Standard diet control (NC), HFHF diet control (HC), HFHF with K2 (K2), and HFHF with K6 (K6). Food intake and body weight were monitored twice per week.

The *L. plantarum* strains ATG-K2 and ATG-K6 were isolated from Korean fermented cabbage that were obtained from AtoGen (AtoGen Co., Ltd., Daejeon, Korea). Each *Lactobacillus* strain was resuspended in phosphate-buffered saline (PBS), and rats were orally administered 5 × 10^8^ CFU of bacteria once a day for 8 weeks.

### 4.2. Preparation of Biological Samples

After 8 weeks, rats were fasted overnight and sacrificed in a CO_2_ chamber. Blood samples were collected and maintained at room temperature for 30 minutes. Plasma was harvested by centrifugation (4000 rpm, 10 min, 4 °C) and stored at –80 °C for future use. The liver, small intestine, and epididymal fat tissues were removed, and their lengths were measured and rinsed with physiological saline (PBS). The ileum section (2 cm) was cut longitudinally and incubated in 2 mL of Dulbecco’s modified Eagle medium (DMEM, CORNING, New York, NY, USA) containing 1% antibiotics and 10% fetal bovine serum (Gibco BRL, Grand Island, NY, USA) in a humidified atmosphere of 5% CO_2_ at 37 °C for 24 h. The supernatants were harvested and stored at − 80 °C until further analysis.

### 4.3. Small Intestine Histology Analysis

A small portion of the ileum tissue was fixed in 10% neutral buffered formalin (Sigma-Aldrich, St. Louis, MO, USA) for paraffin embedding for at least 72 h. Intestinal paraffin sections (3–4 μm) were stained with hematoxylin and eosin (H&E) and observed under an Olympus Provis AX70 microscope (Olympus, Tokyo, Japan) by an experienced pathologist.

### 4.4. Biochemical Measurements

Rat serum and supernatants from the incubated ileum were used for cytokine quantification. Commercial enzyme-linked immunosorbent assay (ELISA) kits (R&D Systems, Minneapolis, MN, USA) were used to determine IL-6 and TNF-α levels, following the manufacturer’s instructions. Epoch Microplate Spectrophotometer (Biotek Inc., Winooski, VT, USA) was used to measure the absorbance.

### 4.5. Quantification of Gene Expression Using Real-Time PCR

Total RNA was isolated from the small intestine tissue using a Total RNA Mini Kit (iNtRON Biotechnology, Seongnam, Korea), according to the manufacturer’s instructions. One microgram of total RNA was used to synthesize the cDNA. Real-time PCR was performed using SYBR^®^ Green Master Mix (TaKaRa Bio, Otsu, Japan) in a QuantStudio3 Real-Time PCR system (Thermo Fisher Scientific, Waltham, MA, USA). The primer sequences (5ʹ-3ʹ) used in the experiments were as follows: FAS forward, GCTGCTACAAACAGGACCATCAC; reverse, TCTTGCTGGCCTCCACTGAC; SREBP-1c forward, CCCTGCGAAGTGCTCACAA and reverse, GCGTTTCTACCACTTCAGGTTTCA; TNF-α forward, GTGATCGGTCCCAACAAGGA and reverse, CTCCCACCCTACTTTGCTTGTG; NF-κB forward, GACCCAAGGACATGGTGGTT and reverse, TCATCCGTGCTTCCAGTGTTT; IL-6 forward, GGGACTGATGCTGGTGACA A and reverse, TCCACGATTTCCCAGAGAACA; β-actin forward, GATTACTGCCCTGGCTCCTA and reverse, TCATCGTACTCCTGCTTGCT. All gene expression levels were normalized to that of β-actin.

### 4.6. Western Blot Analysis for Small Intestine Tissue

Small intestine tissue lysates were extracted using protein lysis buffer (iNtRON Biotechnology, Seongnam, Korea) supplemented with protease and phosphate inhibitors. Equal amounts of protein (20 μg) were separated on 10% polyacrylamide gels and transferred to nitrocellulose membranes (Bio-Rad Laboratories, Richmond, CA, USA). Primary antibodies were purchased from Santa Cruz Biotechnology (Dallas, TX, USA) and Abcam (Cambridge, MA, USA). Blots were developed using ECL western blot detection kit (Amersham Pharmacia, Buckinghamshire, UK) and visualized using the ImageQuantTM LAS500 system (GE Healthcare Life Sciences, Little Chalfont, UK).

### 4.7. Data Analysis

All data are expressed as mean ± SEM. Each experiment was performed in triplicate, and the body weights of the experimental groups were analyzed using GraphPad Prism 5.03 (GraphPad Software, La Jolla, CA, USA) by one-way ANOVA and Tukey’s post-hoc tests. Statistical significance was set at *p* < 0.05.

## Data Availability

Not applicable.
